# Supplementary material for: Workplace Violence Experienced by Personal Care Workers in a District in Seoul, Republic of Korea: A Comparison Study with Office and Service Workers
Source: Healthcare (Basel). 2024 Jan 26;12(3):320. doi: 10.3390/healthcare12030320 (PMC10855380; doi:10.3390/healthcare12030320)
Supplement: Supplementary file 1 [file healthcare-12-00320-s001.zip › healthcare-2772132-supplementary.pdf]

Supplementary material

Table S1. Work-related disease questionnaire

ANSWER ALL

Over the last 12 months, did you have any of the following health problems?

Are your health problems due to your job?

Question	Health problems				Due to your job			
	Yes	No	Don't Know / no opinion	Refused	Yes	No	Don't Know / no opinion	Refused
A) Muscular pains in shoulders, neck and/or upper limbs (arms, elbows, wrists, hands etc.)	1	2	8	9	1	2	8	9
B) Headaches, eyestrain	1	2	8	9	1	2	8	9
C) Anxiety / Depression	1	2	8	9	1	2	8	9
D) Overall fatigue	1	2	8	9	1	2	8	9

ANSWER ALL, IN YOUR MAIN JOB

Over the last month, during the course of your work have you been subjected to any of the following?

Who engaged in such acts?

Question	Act suffered while performing work			
	Yes	No	Don't Know / no opinion	Refused
A) Verbal abuse	1	2	8	9
B) Unwanted sexual attention	1	2	8	9
C) Threats	1	2	8	9
D) Humiliating behaviour	1	2	8	9

ANSWER ALL, IN YOUR MAIN JOB

Over the past 12 months (or since you started your job), during the course of your work have you been subjected to any of the following?

Who engaged in such acts?

Question	Act suffered while performing work			
	Yes	No	Don't Know / no opinion	Refused
A) Physical violence	1	2	8	9
B) Sexual harassment	1	2	8	9
C) Bullying / harassment	1	2	8	9

Table S2. Participants' characteristics before propensity score matching

Characteristics	Personal healthcare workers (n=150)		KWCS					
			Office workers (n=7,063)			Service workers (n=6,983)		
	n	(%)	n	(%)	p-value[a]	n	(%)	p-value[b]
Age, yr								
~54	7	(4.7)	4044	(57.2)	<.0001	4691	(67.2)	<.0001
55-59	12	(8.0)	1162	(16.5)		993	(14.2)	
60-64	36	(24.0)	932	(13.2)		691	(9.9)	
65-69	59	(39.3)	492	(7.0)		338	(4.8)	
70-74	32	(21.3)	247	(3.5)		150	(2.2)	
≥75	4	(2.7)	188	(2.7)		120	(1.7)	
Sex								
Male	9	(6.0)	1641	(23.2)	<.0001	2716	(38.9)	<.0001
Female	141	(94.0)	5424	(76.8)		4267	(61.1)	
Education level								
Below middle school	39	(26.0)	1108	(15.7)	0.0003	517	(7.4)	<.0001
High school graduate	81	(54.0)	3721	(52.7)		3410	(48.8)	
College or more	30	(20.0)	2236	(31.7)		3056	(43.8)	
Monthly income (KRW)								
Less than 2,000,000	109	(72.7)	3141	(44.5)	<.0001	2186	(31.3)	<.0001
2,000,000~3,000,000	32	(21.3)	2313	(32.7)		2311	(33.1)	
More than 3,000,000	9	(6.0)	1611	(22.8)		2486	(35.6)	

KWCS: Korean Working Conditions Survey

[a]P-values between personal healthcare workers and office workers were determined using the Chi-Square test.
[b]P-values between personal healthcare workers and service workers were determined using the Chi-Square test.

Table S3. Odds for psychological or physical violence at work for only female workers

Types of violence	Unadjusted Model			Adjusted Model*		
	OR	(95% CI)	p-value	OR	(95% CI)	p-value
Personal care workers vs. Office workers as reference						
Psychological violence	5.13	(2.99-8.79)	<.0001	4.75	(2.64-8.55)	<.0001
Humiliating behavior	3.01	(1.51-6.02)	0.0018	2.86	(1.35-6.06)	0.0061
Threats	3.22	(1.39-7.46)	0.0065	2.81	(1.14-6.93)	0.0254
Unwanted sexual attention	6.51	(2.79-15.23)	<.0001	7.94	(2.98-21.16)	<.0001
Verbal abuse	4.23	(2.42-7.38)	<.0001	3.75	(2.06-6.83)	<.0001
Physical violence	6.86	(3.60-13.08)	<.0001	5.80	(2.93-11.49)	<.0001
Bullying/harassment	1.00	(0.19-5.01)	0.9930	0.81	(0.13-4.84)	0.8252
Sexual harassment	9.64	(4.37-21.27)	<.0001	7.88	(3.45-18.01)	<.0001
Physical violence	3.49	(1.44-8.48)	0.0054	2.70	(1.04-7.02)	0.0414
Personal care workers vs. Service workers as reference						
Psychological violence	7.01	(3.94-12.50)	<.0001	7.96	(4.04-15.65)	<.0001
Humiliating behavior	3.29	(1.62-6.68)	0.0010	4.24	(1.85-9.71)	0.0006
Threats	3.22	(1.39-7.46)	0.0065	3.60	(1.38-9.41)	0.0091
Unwanted sexual attention	11.65	(4.02-33.77)	<.0001	12.00	(3.37-38.59)	<.0001
Verbal abuse	6.44	(3.47-11.94)	<.0001	6.91	(3.41-14.01)	<.0001
Physical violence	8.94	(4.44-18.01)	<.0001	7.57	(3.45-16.58)	<.0001
Bullying/harassment	1.50	(0.25-9.12)	0.6597	1.16	(0.14-9.72)	0.8893
Sexual harassment	9.64	(4.37-21.27)	<.0001	7.42	(3.16-17.46)	<.0001
Physical violence	25.69	(3.41-193.44)	0.0016	20.35	(2.54-163.32)	0.0046

*Adjusted model was adjusted by participants' characteristics and self-reported work-related symptoms.
